# Engineering skills in the manufacture of tools by wild chimpanzees

**DOI:** 10.1016/j.isci.2025.112158

**Published:** 2025-03-24

**Authors:** Alejandra Pascual-Garrido, Susana Carvalho, Deus Mjungu, Ellen Schulz-Kornas, Adam van Casteren

**Affiliations:** 1 Primate Models for Behavioural Evolution Lab, School of Anthropology and Museum Ethnography, University of Oxford, Oxford, UK; 2 Interdisciplinary Center for Archaeology and Evolution of Human Behaviour, Universidade do Algarve, Faro, Portugal; 3 Department of Science, Gorongosa National Park, Sofala, Mozambique; 4 CIBIO/InBIO, Centro de Investigação em Biodiversidade e Recursos Genéticos, Universidade do Porto, Campus de Vairão, Vairão, Portugal; 5 Gombe Stream Research Centre, the Jane Goodall Institute - Tanzania, Kigoma, Tanzania; 6 Department of Cariology, Endodontology and Periodontology, University of Leipzig, Leipzig, Germany; 7 Department of Human Origins, Max Planck Institute for Evolutionary Anthropology, Leipzig, Germany

**Keywords:** Biological sciences, Zoology, Evolutionary biology

## Abstract

Physical evidence of early hominin perishable tools is scarce. However, it is reasonable to assume the mechanical constraints surrounding tool use and manufacture have remained somewhat constant. Using a functional framework to understand the technical capabilities of extant hominoid tool users presents a novel approach to predict the perishable tool-using capabilities of our earliest relatives. We investigated the structural and mechanical properties of plant materials used by wild chimpanzees to make termite fishing probes. Materials sourced from plant species extensively used by chimpanzees produced implements of greater flexibility than those constructed from plants never selected by chimpanzees. This pattern was also reflected in chimpanzee tool species preferences, with preferred plant species producing highly flexible implements. Implement flexibility aligns with functional predictions and likely facilitates termite attachment. Our findings provide insights into the technical skills associated with perishable artefact-making and raise questions about how this knowledge is learnt and culturally transmitted.

## Highlights

• Materials used by chimpanzees for toolmaking exhibit specific physical properties
• Plant species used for tools produced more flexible probes than plants never used
• Construction of fishing probes suggests an understanding of material properties
• Chimpanzee toolmaking mechanics may inform on the technical skills of early hominins

## Graphical abstract

**Figure:**
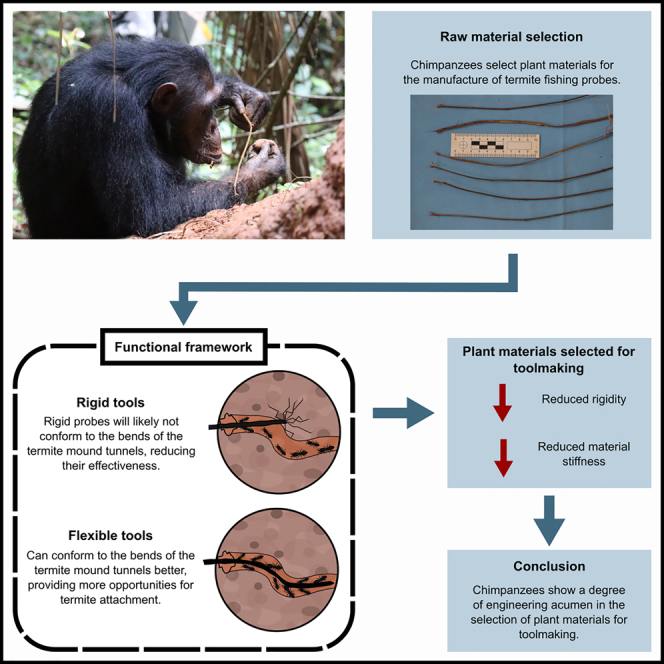

## Introduction

Evidence from even the earliest lithic assemblages suggests that selecting materials based on often inconspicuous mechanical properties has been essential to human tool-using abilities.^1^^,^^2^^,^^3^^,^^4^ For example, Oldowan toolmakers chose finer grained materials more likely to optimally fracture when making stone tools.^1^^,^^4^ Yet, our knowledge of the evolution of toolmaking skills is incomplete, relying on an imperfect archeological record of stone tools.^5^^,^^6^ Comparative data on stone tool use by extant apes and monkeys has been used as a model to fill in some of the gaps left by the archeological record, demonstrating an understanding of the general physical characteristics of the tools used and how this modulates functionality.^7^^,^^8^^,^^9^^,^^10^^,^^11^^,^^12^ However, surprisingly little has been investigated regarding the mechanical characteristics and the resulting biomechanical properties of materials used in toolmaking, with what is known remaining somewhat descriptive^13^^,^^14^^,^^15^^,^^16^^,^^17^ (but see studies by Lamon et al.^18^ and van Casteren et al.^19^). Given their toolmaking abilities,^14^ data from one of our closest living relatives, common chimpanzees (*Pan troglodytes*), are especially valuable.^20^ This is particularly true for the crafting of perishable tools that, by their nature, are generally absent from the archeological record but still remain a significant and highly neglected aspect of technological evolution in the Primate order, including humans.^21^^,^^22^^,^^23^

After humans, common chimpanzees have the most complex and varied repertoires of toolmaking behavior of all extant primate species.^24^^,^^25^ While some populations use stone tools, the majority of chimpanzee tools are sourced from plants^14^^,^^26^ which require an additional manufacturing step for appropriate use.^27^ Given their close phylogenetic proximity to our lineage,^28^ *Pan troglodytes* technical abilities are often compared to the tool using skills of our early ancestors.^3^^,^^6^^,^^8^^,^^14^^,^^29^^,^^30^ Chimpanzees employ tools in a wide range of contexts, including communication, grooming, self-comfort, and protection,^14^ with the most prevalent, complex, and diverse tools used for subsistence.^15^^,^^31^ Implements are manufactured from a diverse breadth of plant materials that vary according to the task.^15^ The wide variety of implements and materials utilized suggests that for certain tasks some materials are better than others, and chimpanzees seem to have an advanced understanding of this.^13^^,^^14^^,^^32^

In early human toolmaking, the mechanical properties of raw materials influenced elements of stone tool production, including invention, spread, and organization of technology.^1^^,^^33^ Likewise, in nonhuman tool production, the mechanical properties of a tool strongly influence the design of the resulting implement and the behavior expressed while using it.^32^^,^^34^^,^^35^^,^^36^^,^^37^ Extensive research on chimpanzee tool use has shown that material selection in tool manufacture is widespread in *Pan troglodytes*.^13^^,^^14^^,^^38^^,^^39^ However, the underlying reasons for this selectivity are not yet fully understood. One possible explanation is that chimpanzees possess a functional understanding of the structural and mechanical properties of the materials used in tool production, which influences their choices. To test this hypothesis, it is necessary to quantify the structural and mechanical properties of both the plant materials selected for tool use and those seemingly overlooked—something that, to our knowledge, has not yet been done in the context of chimpanzee tool use. In this study we investigated the structural and mechanical properties of plant materials that are chosen for the manufacture of termite fishing tools by chimpanzees living at Gombe Stream National Park, Tanzania. While it is well documented^38^^,^^40^^,^^41^ that chimpanzees selectively choose specific material for toolmaking, our research focuses on investigating the underlying physical properties that may influence their choices, a research area that remains largely unexplored.

**Figure 1:**
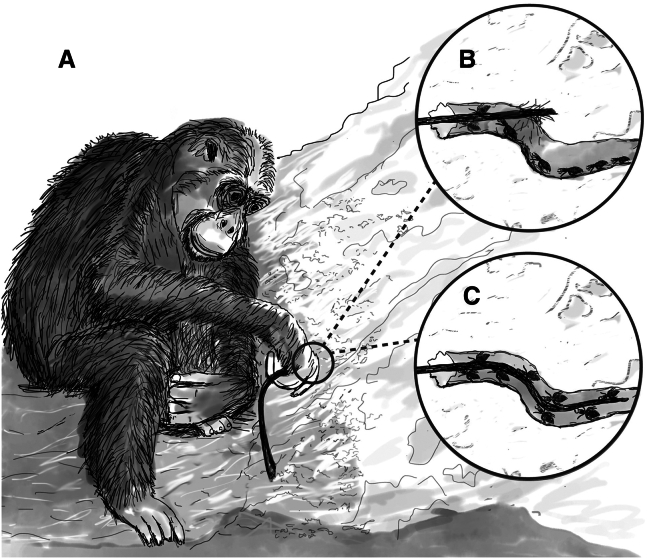
The hypothesized functionality of rigid vs. flexible probes used as termite fishing tools (B and C) When a probe is inserted into a termite mound (A) a rigid probe will likely not conform to the bends of the tunnel reducing its effectiveness (B). A more flexible probe will navigate the tunnels better and likely provide more opportunity for termite attachment (C).

**Figure 2:**
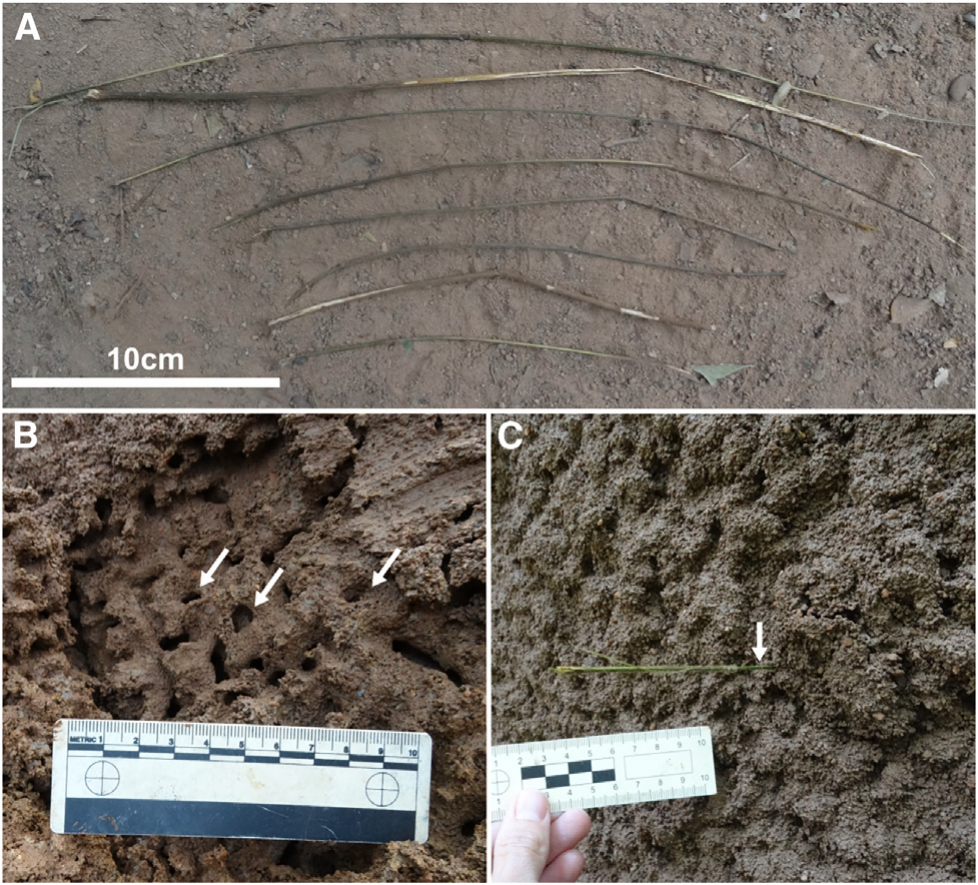
*In-situ* tools used for termite fishing (A) Probe examples of termite fishing tools. (B) White arrows indicate examples of the tunnels found in *Macrotermes* mounds in which the probe tools are inserted. (C) A probe tool left inserted into a *Macrotermes* mound, white arrow highlights the inserted end.

In chimpanzees, termite fishing acumen is acquired primarily through observation of mothers and maternal relatives during the early years of life,^42^^,^^43^^,^^44^ with techniques used varying regionally, including between neighboring communities.^30^^,^^45^^,^^46^^,^^47^ The most common technique, also used by Gombe chimpanzees, consists of the insertion of a single probe into a *Macrotermes* termite exit or alate hole (Video S1). Termite soldiers attack the probe with their mandibles, when the probe is withdrawn the attached insects can be easily consumed^14^ (Figure 1). Probing tools are manufactured from various plant materials (i.e., bark, grass, sedges, twigs, vines, etc.)^14^^,^^40^^,^^45^^,^^46^^,^^48^ sourced from species mostly located near the termite mound, but also occasionally from further away indicating some sort of planful cognitive behavior,^38^^,^^45^^,^^49^^,^^50^^,^^51^ e.g., perception, cognition, and recollection. During processing, implements are generally removed from the main stem and/or lower branches of plants, normally using teeth. The resulting tools at Gombe averaged approximately 28–30 cm (range: 7–118 cm) in length and about 2–4 mm (range: 0.1–7.6 mm) in diameter^45^^,^^46^ (Figure 2A). While part of the variation in tool use techniques and construction materials can be attributed to prey characteristics and ecological context,^30^^,^^32^^,^^40^^,^^45^ others are attributed to social influences that can, if maintained across generations, be termed as cultural.^30^^,^^41^^,^^42^^,^^44^^,^^45^^,^^47^^,^^48^^,^^52^

Video S1. A video of a chimpanzee termite fishing with a plant tool

Chimpanzees at Gombe fish epigeal *Macrotermes* mounds year-round,^45^^,^^46^ though efforts are concentrated during the early wet season (October to December). This timing aligns with the annual reproductive and dispersal cycle of the termites when the insects are more active and accessible. Abandoned tools are regularly recovered by researchers at fished mounds during these months, becoming scarcer during the dry season.^32^^,^^45^ Chimpanzees demonstrate high selectivity regarding the plant material and species used to make their tools, with only a dozen or so species recorded as tool sources from the many more suitable and available plant species found near the termite mounds.^45^^,^^46^^,^^50^ Interestingly, even within known source species, chimpanzees show preferences: some individual plants are used more frequently while others are never utilized.^41^ Given the wide range of inter- and intra-species variation of plant structural and mechanical properties, and that such physical properties could influence the functionality of a constructed tool, we hypothesize that the structural and mechanical properties of plant material may be a selection criterion used by chimpanzees when making their tools.

When probing with beam-like tools an effective implement needs to be flexible; capable of bending to follow the irregular twisting of the termite passages (Figure 1B and 1C; Figure 2B) yet durable enough to maintain its original shape upon withdrawal so as to be reused.^32^^,^^45^ Mechanically, flexural rigidity describes the resistance of a beam,^53^ like probing tools, to bending. It is a salient structural property, encompassing the material a tool is made from and the shape/size of the implement. Measurements of flexural rigidity will effectively quantify the flexibility of a tool. It is expected that tools with low flexural rigidity will be very flexible while tools that demonstrate a higher flexural rigidity will be stiffer and more rod-like. Flexural rigidity is defined as the product *EI*, where *E* is the Young’s or elastic modulus and *I* is the second moment of area.^53^^,^^54^ *E* is essentially the stiffness of a material, its resistance to reversible deformation, measured as the stress (force per unit area) that produces a strain (a proportional change in dimensions).^55^ *E* is a material property; this measurement informs about the material the tool is made from, not how the artifact itself behaves. *I* is the distribution of material around the neutral axis of a beam, essentially describing the shape and size of a tool’s cross section. The *E* of plant tissues can vary within an individual, between members of the same species and interspecifically. This means that plant parts that share similar morphology may behave rather differently to imposed loads, depending on the material they are made up from. The morphology of termite fishing tools is somewhat constrained by the size and shapes of the passages found in epigeal *Macrotermes* mounds^32^ (Figures 2B and 2C); therefore, it seems reasonable to assume that chimpanzees could affect the *EI* of the manufactured tools by choosing materials that deliver desired mechanical behaviors, such as low rigidity.

It has been previously shown in experiments with captive apes (*Pan troglodytes*, *Pan paniscus*, *Pongo pygmaeus*, and *Gorilla gorilla*) that suitable tools can be selected based on rigidity.^56^ However, in these experiments levels of rigidity were not fully quantified and tools were made of unnatural materials that often presented quite stark variation in their levels of rigidity. In natural environments, the variation in the way tools behave mechanically may be much subtler and less conspicuous to the user. The ability of captive apes to select tools with the correct rigidity for a task^56^ and the well-described tool material selection in wild chimpanzees^13^^,^^14^^,^^38^ presents an interesting question. Are wild apes selecting plant materials for tool construction that deliver specific mechanical behaviors in their implements? If this is the case, we would expect chimpanzees to select plant material that produced tools with specific mechanical characteristics.

As stated earlier, termite fishing implements must be flexible to navigate the intricate tunnels of termite mounds. We therefore predict that tools constructed from plant species known to be used as tool sources, would exhibit a lower *EI* than those constructed from plant species never sourced. Similar patterns of low *EI* are to be expected from plant species most preferred for tool construction than less preferred ones. Of those plant species that are known to be used for the manufacture of tools, some individual plants are used regularly by chimpanzees while others are ignored or used rarely.^41^ We predict these popular individual plants should produce tools of a lower *EI* than plants from the same species that have never been used. To test these predictions, we performed flexural rigidity tests on plant materials that are known to be used by chimpanzees in the construction of termite fishing tools and compared these to readily available but unused suitable plant material found close to termite mounds.

## Results

A total of 544 samples obtained from 194 individual plants and 26 species, including sourced and non-sourced species (Dataset S1) were measured and the *EI* and *E* were calculated. The number of samples per species varied, ranging from 3 for *Schrebera alata* to 58 for *Uvaria angolensis*. Samples tested were highly flexible, they had low *EI* values, with data ranging from 0.00010–0.03490 Nm^2^. However, these results are comparable to work conducted on the *EI* of similarly sized stems of sedges.^57^ The plant material our samples were made from demonstrated a wide range of *E*, ranging from 0.105–34.79 GPa, but given the variety of species and materials included, such large ranges are to be expected.^53^^,^^58^ In general, most plant materials tested had a median elastic modulus for each species (range of median elastic modulus = 0.42–28.4 GPa) that was indicative of “woody” plant parts. That is to say, the tissues of a plant that possess lignified secondary cell walls and are generally stiffer than unlignified primary cell walls. In these plant tissues the elastic modulus is proportional to the density of the tissue with values ranging from circa (1–35 GPa).^59^ The one exception to this is bark, with a much lower median elastic modulus compared to other tissues. This is in line with previous bending tests performed on bark that showed a much-reduced stiffness when compared to wood.^60^

### Chimpanzee tool source species

**Figure 3:**
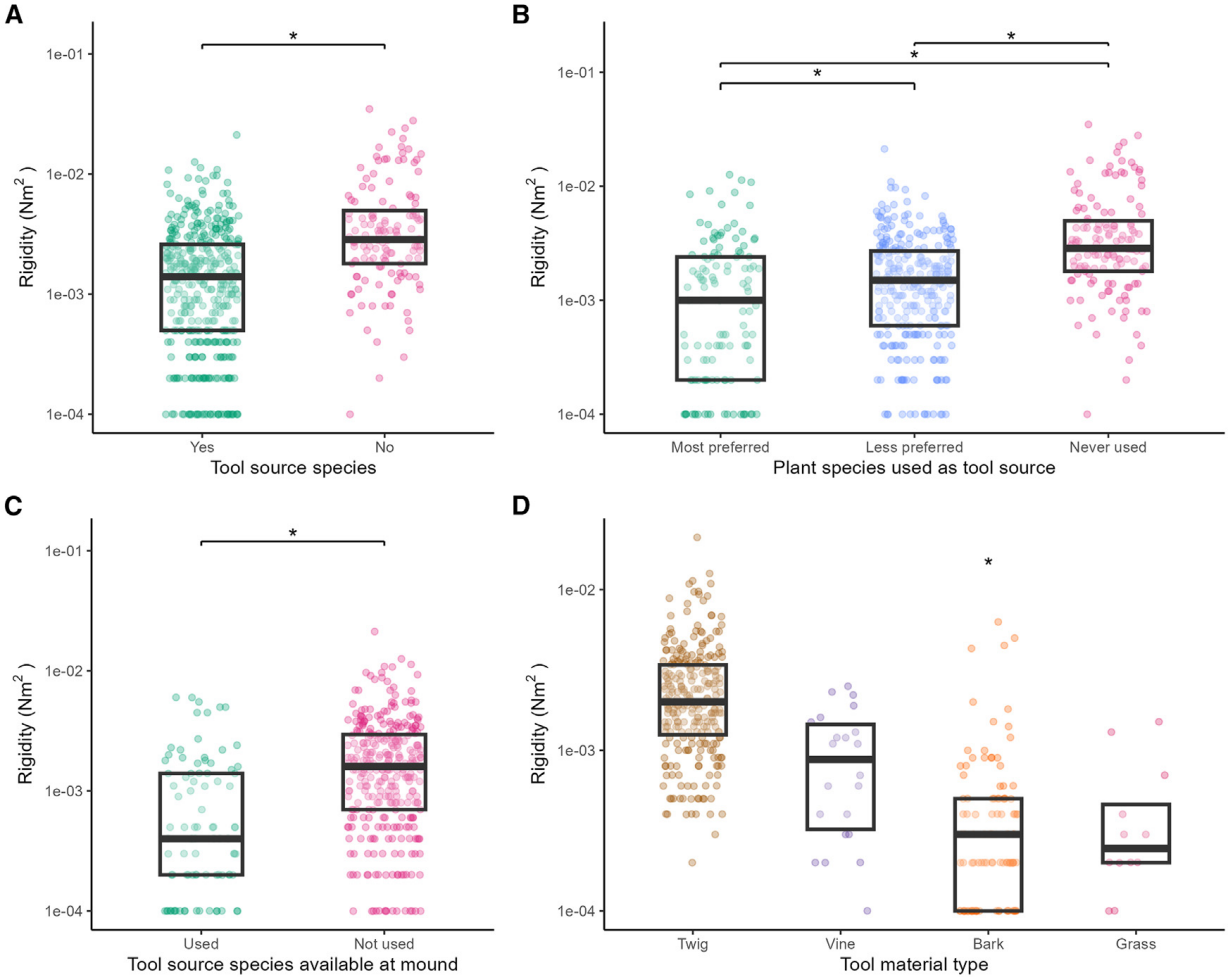
Flexural rigidity (*EI*) of plant material tested (A) Tool source species and non-tool source species. (B) Plant species most preferred, less preferred, and never used. (C) Tool source species available and used and available but never used. (D) Tool material types most frequently used. Black bars represent medians, boxes represent the interquartile range and asterisks represent significant differences between groups (∗ significance level of *p* < 0.05).

**Figure 4:**
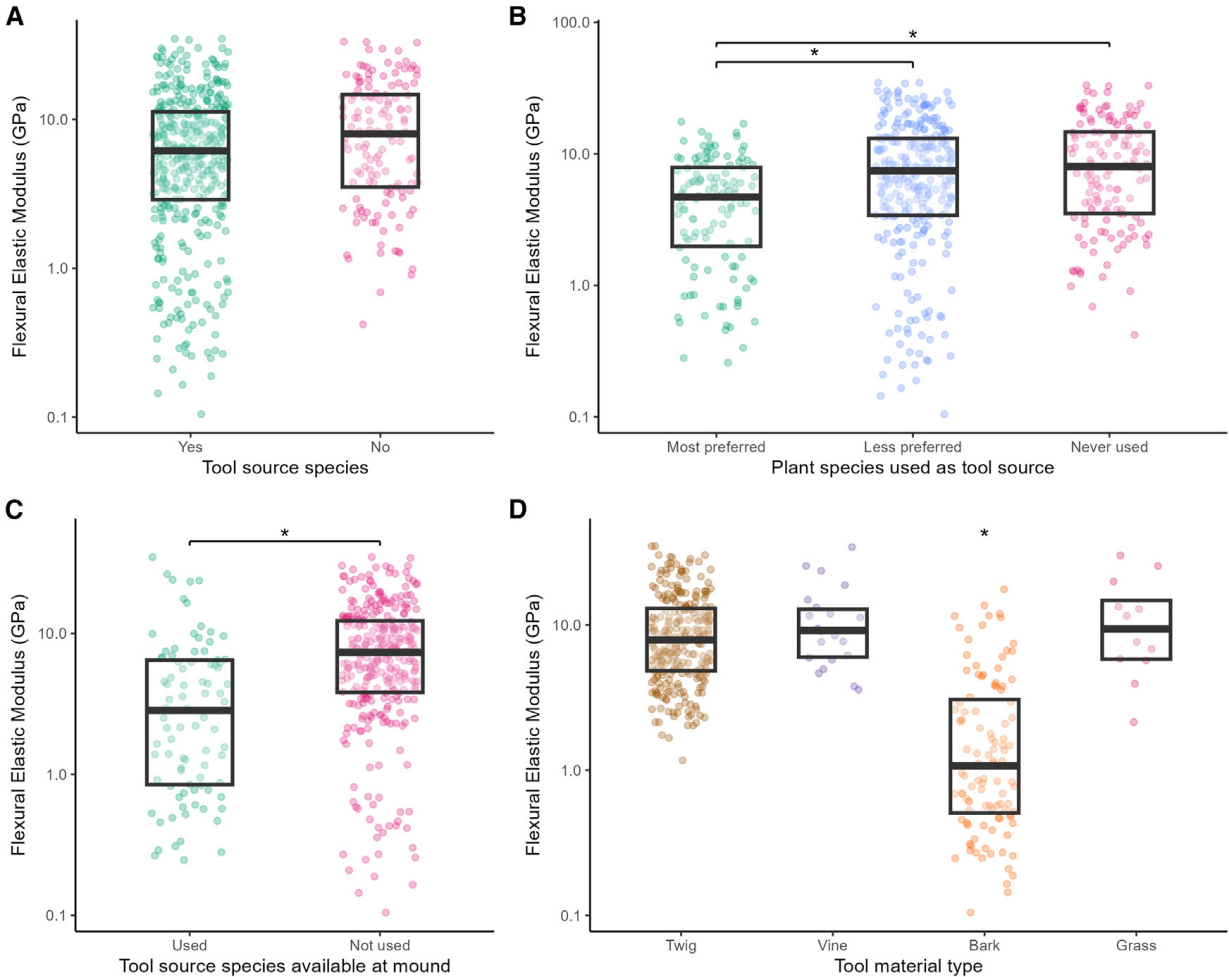
Flexural elastic modulus (*E*) of plant material tested (A) Tool source species and non-tool source species. (B) Plant species most preferred, less preferred, and never used. (C) Tool source species available and used, and available but never used. (D) Tool material types most frequently used. Black bars represent medians, boxes represent the interquartile range and asterisks represent significant differences between groups (∗ significance level of *p* < 0.05).

The results of a linear mixed-effects model revealed that whether an implement was constructed from a plant known to be used as a tool source by chimpanzees had a significant effect on the *EI* of the implement (*F*_(1, 189.84)_ = 14.83, *p* ≤ 0.001) even after accounting for variance in *I* (*F*_(1, 538.69)_ = 266.74, *p* ≤ 0.001) (Figure 3A). This indicates that when controlling for differences in shape and size (i.e., *I*), implements derived from non-tool plant species had a higher *EI* and were therefore more rigid. Post-hoc Tukey-adjusted pairwise comparisons confirmed this difference (t = −3.85, *p* ≤ 0.001) with implements manufactured from non-tool plant species shown to be more rigid. This relationship does not hold true for flexural elastic modulus. Although the results of our linear mixed effect model suggested that non-tool sources may possess a slightly higher elastic modulus there was no significant difference between the groups (*F*_(1, 187.17)_ = 2.55, *p* = 0.112) (Figure 4A). A plant species’ status as a tool source does not appear to reliably predict elastic modulus of the plant tissue.

### Chimpanzee individual plant and plant species preference

Interestingly, within known tool source species, whether an individual plant had tool removals/scars as a result of having been sourced by chimpanzees, seemingly had a significant effect on *EI* (*F*_(1, 165.76)_ = 22.98, *p* ≤ 0.001) even when accounting for the variance in *I* (*F*_(1, 404.66)_ = 187.5, *p* ≤ 0.001) (Figure 3C). This pattern also held true for *E* (*F*_(1, 178.11)_ = 11.28, *p* ≤ 0.001) (Figure 4C). Post-hoc Tukey adjusted pairwise comparisons confirmed this difference with plants actually used by chimpanzees having significantly lower *EI* (t = −4.79, *p* ≤ 0.001) and *E* (t = −3.36, *p* = 0.001) than those plants of known tool sources that showed no overt signs of use by chimpanzees. This indicates that plants regularly used by chimpanzees to construct tools produce more flexible tools that are made of material with a reduced stiffness.

Flexible tools were also associated with tool species preference. Based on previous research that demonstrated tool material and plant species selectivity in the Gombe chimpanzees,^41^^,^^46^^,^^50^ we ranked species as “most preferred” for tool species most frequently represented in tool assemblages (i.e., 11.7% or more from the total of tools recovered); as “less preferred” for species represented less frequently in tool assemblages (i.e., 10% or less from the total of tools recovered); and as “never used” for species available near the mound but never recorded as tool sources. Linear mixed models demonstrated that chimpanzee preference for certain plant species for the construction of their tools had a significant impact on both the *EI* (*F*_(2, 190.58)_ = 16.44, *p* ≤ 0.001) even when accounting for *I* (*F*_(2, 534.35)_ = 272.36, *p* ≤ 0.001) (Figure 3B) and *E* (*F*_(2, 188.86)_ = 5.83, *p* ≤ 0.01) (Figure 4B). Post hoc analysis indicated that most preferred tool source species (i.e., *Monanthotaxis poggei*) had significantly lower *EI* and *E* when compared to less preferred tool source species (*EI*: t = −4.11, *p* ≤ 0.001; *E*: t = −3.00, *p* ≤ 0.01) or never used species (*EI*: t = −5.63, *p* ≤ 0.001; *E*: t = −3.06, *p* ≤ 0.01). While there was a significant difference between the *EI* of less preferred tool source species and never used plant species (t = −2.50, *p* ≤ 0.05) this difference was not borne out in the material that these implements were made out of as there was no significant difference between the *E* of these groups (t = −0.572, *p* = 0.835).

### Raw material type

Linear mixed-effects models revealed the influence that raw material type had on chimpanzee tool material choice. Raw material type significantly influenced *EI* (*F*_(3, 133.45)_ = 112.04, *p* ≤ 0.001) even after controlling for *I* (*F*_(1, 351.82)_ = 218.40, *p* ≤ 0.001) (Figure 3D). Post hoc comparisons indicated that implements made from bark exhibited significantly different *EI* compared to those made from twigs (t = 18.32, *p* < 0.001), vines (t = 6.35, *p* < 0.001), and grass (t = −4.29, *p* ≤ 0.001). No other pairwise comparisons showed a significant difference.

Similarly, when examining *E*, a linear mixed-effects model also indicated a significant effect of raw material type (*F*_(3, 134.07)_ = 46.98, *p* < 0.001) (Figure 4D). Post hoc comparisons showed that bark had a significantly lower *E* compared to twigs (t = 11.60, *p* ≤ 0.001), vines (t = 5.35, *p* ≤ 0.001), and grass (t = −4.54, *p* ≤ 0.001). Differences among twigs, vines, and grass were not statistically significant. Together, these results suggest that the use of bark, consistently produces flexible implements with lower *EI* that are constructed from a material of lower *E*, compared to those made from twigs, vines, or grass.

## Discussion

This study is the first to examine the structural and mechanical properties of raw material used for toolmaking in a wild nonhuman ape species to elucidate the selection criteria behind their choice. Our results indicate that the mechanics of plant tissues are a factor in selecting materials for the construction of termite fishing probes by Gombe chimpanzees, notably low *EI* and reduced *E*. The selection of tool materials based on specific physical properties has already been described in the use of stone tools by nonhuman primates.^7^^,^^8^^,^^11^^,^^12^^,^^61^ Our research therefore extends the technological knowhow of wild chimpanzees to their toolmaking behavior.

Using appropriate raw materials is essential for functional tool use and performance.^14^^,^^35^^,^^62^^,^^63^ Failure to do so will likely affect the efficiency and durability of the tool and, consequently, the performance of the tool-user and the energetic payoffs of its use.^18^^,^^64^ When probing with beam-like tools, such as those crafted for termite fishing, the *EI* of the implement will be instrumental to its effectiveness. Our results indicate that chimpanzees at Gombe do routinely manufacture tools that display specific structural and mechanical properties that could facilitate efficient task completion. Plant species known to be tool sources produced implements that were 51% less rigid (Figure 3A) than implements crafted from non-tool source species. Our results indicate that the previously observed choice of plant materials by chimpanzees^41^^,^^46^^,^^50^ makes sense mechanically. More flexible implements will bend more easily circumventing the need for high forces when pushing it into *Macrotermes* tunnels, and such implements would be better at curving around the bends, rather than puncturing the walls and getting stuck within the mound (Figure 1). Interestingly, such seemingly mechanistic tool choices by Gombe chimpanzees mimic what previous researchers had predicted from termite fishing observations (Video S1).^32^^,^^45^ Human observers hypothesized that termite fishing probes must be flexible and resilient if they are to yield many termites^32^^,^^45^ (Figure 1B)—here we show that chimpanzees seem to share such technical understandings.

Low *EI* was also associated with plant species preferred for the construction of termite fishing tools (Figure 3B). Most preferred plant species (i.e., *Monanthotaxis poggei*^46^) produced implements that were substantially more flexible than other available plants, with less preferred species being 12% more rigid, and never used species being 175% more rigid. Interestingly, preferred source species by Gombe chimpanzees, such as *Grewia forbesii* and *Uvaria angolensis*,^46^ are also the favorite tool material chosen by chimpanzee communities living elsewhere in Tanzania: at Mahale and the Issa Valley.^46^^,^^49^^,^^50^ Furthermore, *Grewia* spp. provides tool material for termite fishing apes living more than 5,000 km away: at Mt. Assirik^40^ and Fongoli in Senegal.^65^ The maintenance of preferred plant species across vast geographical ranges is somewhat perplexing given the variety of other suitable plants in these biomes. Unless other microecological variables, such as the abundance of raw material, influence this choice, it is highly possible that the structural and mechanical suitability of these plant species for termite fishing probes will have contributed to the ubiquity of their selection. Mapping the mechanical commonalities in perishable tool materials may be a novel lens to study knowledge transmission and culture in chimpanzees. The ubiquity and variety of perishable tool use across the chimpanzee range offers a broad dataset to unravel the emergence and diffusion of such behaviors, especially when compared to the smaller geographical range of *Pan* lithic technologies. Technical skills may be obtained through trial-and-error learning but social learning may also contribute via stimulus enhancement or active facilitation (tool sharing).^43^^,^^44^ Future studies should focus on disentangling the mechanisms enabling the understanding of tool mechanics and the dissemination of this knowledge.

But even within source species, not all individual plants are used by Gombe chimpanzees.^41^ This could be the result of inter-individual differences that may influence their aptness as a tool source. The finding that among source species, individuals that have been procured by chimpanzees for tools provided material that was significantly more flexible and of a lower stiffness compared to equivalent individuals that show no overt signs of use by chimpanzees (Figures 3C and 4C) supports this assertion. Future systematic studies are necessary to properly assess intra-and inter-individual differences on raw material properties and elucidate how this relates to tool material choice by apes.

We found that plant species that were favored by Gombe chimpanzees for the manufacture of flexible termite fishing probes^50^ had a significantly lower material stiffness (Figure 4). This is interesting as the stiffness of a material is not a very salient property and cannot be assessed by how a tool behaves unlike *EI*. It is possible though that low *E* could be selected for by proxy instead. Choosing a material with lower *E* permits the construction of an implement with a larger cross-sectional area and/or a different cross-sectional shape that can still exhibit the very low *EI* required to navigate the tunnels of a termite mound. In our study, bark produced some of the most flexible implements (Figure 3D) and had a significantly lower *E* than any of the other tool materials tested (Figure 4D). Bark is a well utilized termite fishing tool material, and for some chimpanzee communities living in drier, more open miombo woodland habitats, the only material used.^49^ There is some evidence that bark tools are morphologically different from tools made from other materials. Bark and twig tools recovered at Mahale (Bilenge) were the same length but tools made from bark were significantly wider.^48^ A similar finding was recorded in previous studies at Gombe where the use of bark resulted in significantly wider tools.^46^ It is a possibility that such tools could offer a larger or more varied surface area for termite attachment and therefore increase termite acquisition. In this way plant species that have woody tissues that generally exhibit a lower *E*, such as bark, may be preferred as they offer a trade-off between axial flexibility and functionally optimal cross-sectional shapes. Our data appears to suggest this could be the case (Figure 4).

Analyzing how the structural and mechanical properties directly influence tool efficiency was beyond the scope of the present paper, but such analysis will be crucial to better understand the selection criteria behind the material used to construct tools. It is possible that other non-mechanical factors also contribute to tool material choice. Certain materials may require a shorter time of searching or manufacture, making it less costly to produce and thus saving effort when harvesting a food source.^18^^,^^30^^,^^51^ Likewise, tool material choice may be a response to the specific structural characteristics of the termite mound, such as size and thickness, or the behavior of the termites as prey.^30^^,^^66^ Without more widespread data on the structural and mechanical properties of chimpanzee tools it is hard to understand the extent to which mechanics influences material selection in chimpanzees when compared to other non-mechanical factors.

The ability of chimpanzees to discriminate materials for toolmaking with often highly specific structural and mechanical properties, as shown in this study, may be a potentially vital component of their remarkable tool-related skills. That the use of tool materials and source species, as well as the combination of technical elements is community-specific,^15^^,^^40^^,^^45^^,^^47^^,^^49^ implies that their learning processes result in consistent outcomes. Termite fishing is a complex tool-aided foraging behavior acquired by chimpanzees during their early years of life, with mothers being the primary model for learning.^42^^,^^44^^,^^67^ Individual experimentation with different materials through trial and error accompanied by various forms of social learning may likely influence the acquisition of choice of materials for construction.^42^^,^^43^^,^^44^^,^^67^^,^^68^ At Gombe, infants reused abandoned tools and took tools from others.^44^ Female offspring watch their mothers more compared to male offspring and are more similar with respect to the length of the tool inserted into the termite nest.^67^ These observations suggest that multiple social learning mechanisms occur in this context, including stimulus enhancement, peering observation or active facilitation (i.e., tool transfers). Future work investigating the ontogeny of toolmaking, including individual use and/or preference for tool materials and across matrilines,^69^ will contribute to understand how much of the individual and/or social learning contributes to variation in toolmaking within and between chimpanzee populations.

Since the initial discoveries of their outstanding tool use abilities, chimpanzees have provided invaluable insights for understanding the emergence, maintenance and behavioral diversity in primate material culture.^8^^,^^11^^,^^14^^,^^15^^,^^18^^,^^26^^,^^30^^,^^38^^,^^41^^,^^42^^,^^44^^,^^47^^,^^70^^,^^71^ Regional variation in chimpanzee technologies have so far been attributed to ecological explanations,^30^^,^^40^^,^^45^^,^^72^ the physical constraint imposed by the intended task,^13^^,^^32^ tool efficiency,^18^^,^^64^^,^^73^ the characteristics of the insect prey,^30^^,^^74^ and social influences.^11^^,^^41^^,^^44^^,^^47^^,^^71^^,^^72^ Our study cautions that plant material properties may also be responsible for dictating between-population variation in chimpanzee technology. Thus, when assessing the material culture in wild apes and other non-human primates, it is essential to chart the availability and usage of different plant species, as well as their mechanical properties. Investigating this will not only provide a valuable window into the cognitive skills associated with the production of perishable tools, a largely overlooked topic in human technological evolution,^5^^,^^21^ but will contribute to understanding the factors shaping toolmaking habits in wild ape populations. This information may, in turn, have implications for interpreting the technical abilities of hominin tool makers,^1^^,^^4^ and might be especially interesting to shed some new light on the evolution of wood processing and cultural development of wood technologies, how they are learnt and culturally transmitted. Oldowan hominins were selecting materials based on their often inconspicuous mechanical properties for manufacturing cutting tools, such as raw materials more apt for flaking.^1^^,^^4^^,^^75^ Likewise, recent evidence demonstrates raw material selection grounded on mechanical properties (i.e., hardness, elasticity) for the manufacture of wooden tools by Pleistocene hominins.^23^ Based on the toolmaking abilities described in this study for one of human’s closest living relative with perishable implements, it is reasonable to assume that such skills extended beyond the use of lithic technologies, and were therefore likely more ancient, diverse, and complex than currently depicted by the human lithic archeological record.^1^^,^^4^^,^^76^

### Limitations of the study

While this study provides the first comprehensive evidence that the mechanics of plant tissues is a factor in raw material choice for toolmaking by wild chimpanzees, how this factor contributes to tool material selection by, for example, improving tool efficiency has not been analyzed. Furthermore, it is possible that other non-mechanical factors such as a shorter time of tool material searching, social influences, or characteristics of the termite mound will also contribute to preferential use of tool material by wild chimpanzees. Moreover, different communities may use different techniques that may make one plant material or species more effective. Incorporating such a holistic approach to the study of raw material procurement by wild chimpanzees will be fundamental to fully comprehend the factors that have led to the emergence and diversity of chimpanzee plant-based material cultures.

In addition, in our mechanical analysis we have used regular shapes to calculate the second moment of area, this was necessitated by the logistics of the project. However, this undoubtedly induced some level of error into the results. A more detailed shape analysis possibly using digital methods and/or incorporating computational modeling may reveal finer scale differences in the structural and/or mechanical properties of termite fishing implements. We also could not actually sample the tools created by chimpanzees because of methodological difficulties. If such difficulties were surmountable, measuring the *EI* and *E* of actual tools might reveal functional or cultural foundations to plant material selection not captured by the plant material proxies used in this study.

## Resource availability

### Lead contact

Requests for further information and resources should be directed to and will be fulfilled by the lead contact, Adam van Casteren (adam.vancasteren@gmail.com).

### Materials availability

This study did not generate new unique reagents.

### Data and code availability

• Data analysis was performed in R using standard packages. Code chunks used in the data analysis in this study can be found in the supplementary data (Data S1–S8). All data used in this analysis is available in the manuscript or the supplementary dataset.
• Raw data files can be found at DOI: https://doi.org/10.5281/zenodo.14771245.
• Any additional information required to reanalyze the data reported in this paper is available from the lead contact upon request.

## Acknowledgments

We thank the John Fell Fund University of Oxford and the Dennis Stanfield Memorial Fund granted by the 10.13039/501100001264Linnean Society of London for funding this research. A.P.-G. was supported by The Leakey Foundation during the writing stage of the manuscript. We are grateful for permission to conduct research in Gombe Stream National Park by Tanzania National Parks (TANAPA), Tanzania Wildlife Research Institute (TAWIRI), and Tanzania Commission for Science and Technology (COSTECH); field assistance by Nuhu Japhary Buke; critical logistical support by The Jane Goodall Institute (Tanzania); and plant species identifications by Dr. Frank Mbago, Department of Botany, University of Dar es Salaam. Special thanks go to Dr. Anthony Collins and Herman Dondidondi for valuable support with carrying out this research and for aiding logistics in the field. We thank Prof. Roland Ennos for reading and commenting on an early version of the manuscript and three anonymous reviewers for their helpful comments on the manuscript.

## Author contributions

Designed research, A.P.-G., S.C., E.S.-K., and A.v.C.; collected data, A.P.-G.; analyzed data, A.v.C.; writing – original draft, A.P.-G. and A.v.C.; writing – review & editing, A.P.-G., S.C., D.M., E.S.-K., and A.v.C.

## Declaration of interests

The authors declare no competing interest.

## STAR★Methods

### Key resources table

**Table:** 

REAGENT or RESOURCE	SOURCE	IDENTIFIER
**Deposited data**		

Raw data - Force and Displacement	This study	DOI: https://doi.org/10.5281/zenodo.14771245
Data - *E*	This study	N/A
Data - *EI*	This study	N/A
Chimpanzee tool material preference	Pascual-Garrido & Almeida-Warren^41^; Pascual-Garrido^46^; Pascual-Garrido^50^	N/A

**Software and algorithms**		

Program R version 4.3.1	https://www.r-project.org/	N/A

### Experimental model and study participant details

A total of 544 samples of plant tissue were obtained from 194 individual plants representing 26 species (detailed in Dataset S1).

#### Study site

Our study took place at Gombe Stream National Park in west Tanzania (S 4.67, E 29.65; 766-1,623 m.a.s.l.). The 35km^2^ park, set on the eastern shore of Lake Tanganyika, is characterized by deep valleys which fall from the rift escarpment to the Lake. The valley bottoms are dominated by evergreen forests, woodlands cover the slopes, and ridges are carpeted with grasslands. We focused on the habituated Kasekela community which resides in the centre of the park and whose individuals have been studied for more than 60 years, remaining the longest wild chimpanzee community ever to be studied.^77^^,^^78^

#### Sample collection

Data were collected by A.P.-G assisted by an experienced Tanzanian research assistant over 90 days, from 02 June 2021 to 31 August 2021. Samples were collected from a total of nine *Macrotermes* mounds that are habitually fished by the apes and labelled with a unique identifier (GTMXXX). A mound was considered to be fished if one or more of the following criteria applied: 1) chimpanzees were seen fishing, 2) presence of artefacts, fragments, or debris resulting from the process of manufacture, 3) presence of scars on plants near the mound because of apes removing material for tool manufacture.^50^

Termite fishing tools are normally reused during a termite fishing bout before being abandoned by the apes.^14^^,^^45^ Once abandoned, it is not possible for the researchers to recover the tools until all the individuals from the fishing party have left the mound, which can last from some minutes up to more than four hours.^79^ It is unknown whether tools are abandoned because feeding has finished or because the functionality of the tool has diminished. However, factors like drying and repeated use could be responsible for changes in the structural and mechanical properties of tools over such extended time periods. To limit such effects and to most accurately represent the mechanical properties of a tool material when chosen by a chimpanzee, all samples were collected from living plants.

Previous data was used to inform tool material and plant species.^45^^,^^46^^,^^50^ Similarly, extensive work conducted on the morphology of termite fishing tools at Gombe was used to inform the dimensions of samples collected for four-point bending (see below).^41^^,^^46^^,^^50^ For plant species that provide more than one type of raw material (*i.e., Monanthotaxis poggei* provides bark and twig material^50^), all material types were collected. To compare the structural and mechanical properties of source species with non-source ones, individuals from non-source species were also sampled. Given that Kasekela apes utilize tool material mostly close to the termite mound,^45^^,^^50^ samples were obtained from a selected number of individual plants located within a 20 m radius from the center of the mound from which suitable raw material for termite fishing probes (i.e., long thin pieces of plant material) could be obtained by the researcher using the hand or fingernails, including source and non-source species. For each individual plant, three randomly selected samples were collected. This involved the researcher removing plant parts (*i.e.,* bark, twig, vine, grass) that could easily be detached with hands or fingernails from locations normally sourced by chimpanzees (*i.e.,* main stem and/or lower branches)^49^^,^^50^ and within dimensions reported for termite fishing tools at Gombe.^45^^,^^46^ Once removed from the mother plant, samples were immediately placed into a Ziploc bag which contained 250 ml of water to avoid desiccation. For each sample the following parameters were recorded: Mound identifier (GTMXXX), GPS location, plant species, part of plant sampled (*i.e.,* branch, stem), and raw material type (*i.e.,* bark, twig, grass, vine). Given that even within source species, not all individuals are used,^41^ it is possible that inter-individual differences on their mechanical properties make some specimens more suitable than others. To test for this, the presence of removals/scars^50^ was taken as evidence of the specimen being sourced by the apes for tool material in the past. Each sample was returned to camp where its length (cm), breadth (mm), and depth (mm) or diameter (mm) if circular in cross section, were measured using an electronic digital caliper. Four-point bending tests were performed on each sample within 3 hours after collection. For plant species known by the researchers, the species name was recorded at time of sample collection. For plant species unknown, a sample of the individual plant including leaves, stalks, fruits, and/or flowers (when available) was collected and curated in camp for later identification by Dr. Frank Mbago, Department of Botany, University of Dar es Salaam.

### Method details

#### Mechanical testing

Four-point bending tests were used to measure the *EI* and *E* of the samples collected. During four-point bending a beam-like sample is bent between two supports by lowering two central points onto a sample from above, placing the structure into pure bending (Figure S1). Due to the pure bending, this method does not require any specific span to depth ratio as necessitated by three-point bending. The *EI* of the sample can then be calculated using Equation 1.^53^^,^^54^

(Equation 1) $$EI=\frac{dF}{dx}\frac{a}{48}\left(3L^{2}-4a^{2}\right)$$

Where *dF/dx* is the initial slope of a force displacement curve (Figure S1C), *L* is the distance between the lower supports and *a* is the distance between inner and outer probes (Figure S1C).

Dividing *EI* by *I* gives an estimation of *E* for a given tool. We calculated *I* by assuming regular cross sections of samples. Plant parts rarely exhibit perfectly regular shapes so this assumption would induce some estimation error into our dataset. However, such assumptions have been utilized in previous studies^19^ and the induced error would be uniform across all data. If the estimated *E* appeared erroneously high (>35GPa)^59^ the sample was removed from the dataset. Samples were classed as either having a circular cross section or a rectangular cross section and *I* was calculated using Equation 2 for circular cross-sections,^53^ where *R* is the radius of the tool.

(Equation 2) $$I=\frac{\pi R^{4}}{4}$$

*I* was calculated for rectangular cross sections using Equation 3, where W is the width of a sample and D is the depth.^53^

(Equation 3) $$I=\frac{WD^{3}}{12}$$

All four-point bending tests were conducted on a portable universal testing machine (FLS-1, Lucas Scientific, Panama). This equipment consisted of a hand-cranked movable crosshead with a force transducer to measure the resultant forces and a linear variable displacement transducer that accurately measured movements in the crosshead. The tester is powered by and interfaces with a laptop computer upon which custom built software for the collection of force/displacement data.^54^

### Quantification and statistical analysis

All statistical analysis was carried out using the Program R version 4.3.1. All data used in statistical analysis can be found in Dataset S1 and full statistical table outputs can be found Supplementary Data (Data S1–S8). We set the p threshold as 0.05 for all our statistical tests.

#### Comparing *E* and *EI*

Similar procedures were used when comparing *E* and *EI*. Samples were grouped based on previous research on the selectivity of plant material for termite fishing tools at Gombe Stream National Park.^45^^,^^46^^,^^50^

The data set was variously subsetted for analysis. Firstly, data was separated into plant species that were used as tool sources or not Category: “Tool source species” groups: “yes” or “no” (Figures 3A and 4A, “yes” n =408, “No” n = 136;). Previous research had indicated finer-scale preferences within the plant species used as source material for termite fishing tools.^45^^,^^46^^,^^50^ To reflect these preferences, we further subdivided the "yes" group into two categories with the following data split Category: “Plant species used as tool source” groups: “Most preferred”, “Less preferred” and “Never used” (Figures 3B and 4B, “Most preferred” n = 122, “Less preferred” n = 286, “Never used” n = 136).

To test for difference in individual plant preference the data set was subdivided based on the actual plants the material was sourced from. Non-tool source species were removed from the dataset. The remaining samples were categorized by whether a plant showed salient removals/scars produced by chimpanzees when using a particular plant as a tool source^50^ or not. Category: “Tool source species available at mound” groups: “Used”, “Not used” (Figures 3C and 4C, “yes” n = 89, “No” n = 319).

Finally, to understand the variance in structural and mechanical properties in relation to raw material type, samples were visually assigned to broad material types. Categories: “Tool material type” groups: “Twig”, “Vine”, “Bark”, “Grass” (Figures 3D and 4D, “Twig” n = 267, “Vine” n = 22, “Bark” n = 107, “Grass” n =12).

For statistically comparing *EI* and *E* we used Linear Mixed Effect Models (LMM) using the “lm4e” package in R.^80^^,^^81^ This was done in order to account for covariates such as fixed effect variables (continuous and categorical) and random effects. This is especially important when analyzing *EI*, as this property is the product of *E* (material stiffness) and *I* (second moment of area). By including *I* as a continuous fixed effect, the model controls for geometric differences between tools. This allows us to isolate whether differences in *EI* are related to the various subsetted categories and not just an artifact of implement geometry. For both *EI* and *E* we included a random effect variable of plant to account for multiple samples being collected from any one individual plant. As *E* is a mechanical property it is already geometrically normalized by its nature. This meant that for analyses involving *E* we dropped the continuous fixed effect of *I* and retained the various categorical fixed effects. An ANOVA (type II) was run using the “lmerTest” package in R^80^^,^^82^ followed by post-hoc analyses comparing estimated marginal means (EMMs) was performed to conduct pairwise comparisons between the categorical fixed independent variables using the “emmeans’ package.^80^ To ensure that the residuals of our models were normally distributed *EI* and *E* values (and *I* where applicable) were log-transformed. Full models and outputs can be found in Supplementary Data (Data S1–S8).

## Supplemental information

Document S1. Figure S1 and Data S1–S8

Dataset S1. The complete dataset used in analysis
