# Supplementary material for: Engineering skills in the manufacture of tools by wild chimpanzees
Source: iScience. 2025 Mar 24;28(4):112158. doi: 10.1016/j.isci.2025.112158 (PMC12131256; doi:10.1016/j.isci.2025.112158)
Supplement: Document S1. Figure S1 and Data S1–S8 [file mmc1.pdf]

iScience, Volume *28*

Supplemental information

Engineering skills in the manufacture of tools by wild chimpanzees

Alejandra Pascual-Garrido, Susana Carvalho, Deus Mjungu, Ellen Schulz-Kornas, and Adam van Casteren

Supplementary Figures

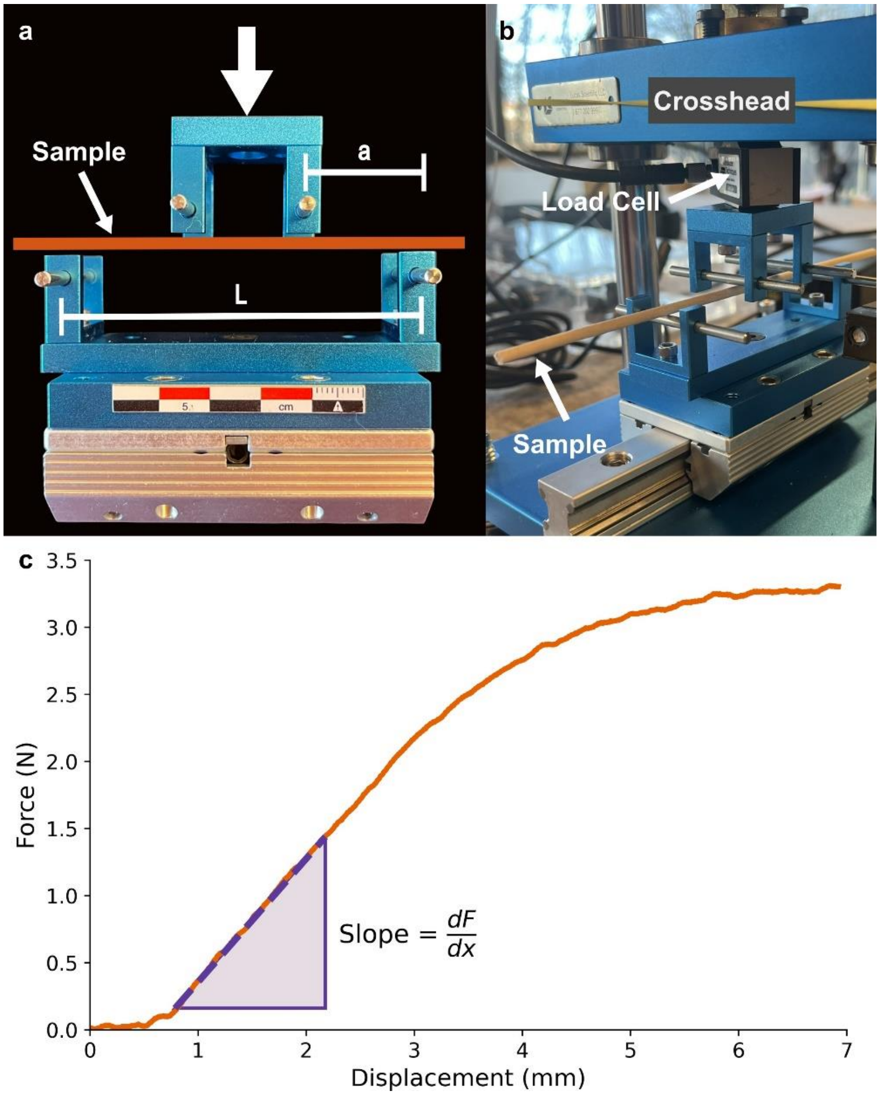

Figure S1. The four-point bending method for beam like samples. (a) Four-point bending, where a beamlike sample is supported by two lower supports and two points of contact are lowered onto the sample placing it into bending. (b) The four-point bending set up on the portable universal tester, as used in this study. (c) Force and displacement data generated from placing a termite fishing probe into four-point bending. The slope of the initial linear region of this plot is used in equation 1 to calculate *EI* and *E*.

Supplementary Data

Data S1: Full LMM model output comparing whether chimpanzee plant species preference (preference) is driven by *EI*, related to Figure 3b and STAR Methods.

```
# Preference model
pref_model= lmer(log10(EI) ~ preference + log10(I) +(1|plant), data=data)
summary(pref_model)

## Linear mixed model fit by REML. t-tests use Satterthwaite's method [
## lmerModLmerTest]
## Formula: log10(EI) ~ preference + log10(I) + (1 | plant)
##    Data: data
##
## REML criterion at convergence: 341
##
## Scaled residuals:
##     Min      1Q  Median      3Q     Max
## -3.0372 -0.3925  0.0331  0.4125  4.2993
##
## Random effects:
##  Groups   Name        Variance Std.Dev.
##  plant    (Intercept) 0.12493  0.3535
##  Residual             0.05241  0.2289
## Number of obs: 544, groups:  plant, 194
##
## Fixed effects:
##              Estimate Std. Error        df t value Pr(>|t|)
## (Intercept)   3.93154    0.43420 531.49401   9.055  < 2e-16 ***
## preferenceL   0.27531    0.06706 190.81740   4.105 5.98e-05 ***
## preferenceN   0.44702    0.07938 192.22347   5.631 6.28e-08 ***
## log10(I)      0.55982    0.03392 534.35259  16.503  < 2e-16 ***
## ---
## Signif. codes:  0 '***' 0.001 '**' 0.01 '*' 0.05 '.' 0.1 ' ' 1
##
## Correlation of Fixed Effects:
##             (Intr) prfrnL prfrnN
## preferenceL -0.113
## preferenceN -0.243  0.571
## log10(I)     0.992 -0.009 -0.156

# Preference model post-hoc
anova(pref_model, type = 2)

## Type II Analysis of Variance Table with Satterthwaite's method
##             Sum Sq Mean Sq NumDF  DenDF F value    Pr(>F)
## preference  1.7237  0.8618     2 190.58  16.444 2.582e-07 ***
## log10(I)   14.2745 14.2745     1 534.35 272.361 < 2.2e-16 ***
## ---
## Signif. codes:  0 '***' 0.001 '**' 0.01 '*' 0.05 '.' 0.1 ' ' 1
```

```r
emmeans(pref_model, list(pairwise ~ preference), adjust = 'tukey', lmer.df =
'satterthwaite')
```

```
## $`emmeans of preference`
##  preference emmean     SE  df lower.CL upper.CL
##  M           -2.89 0.0577 213    -3.01    -2.78
##  L           -2.62 0.0417 225    -2.70    -2.54
##  N           -2.45 0.0560 183    -2.56    -2.34
##
## Degrees-of-freedom method: satterthwaite
## Results are given on the log10 (not the response) scale.
## Confidence level used: 0.95
##
## $`pairwise differences of preference`
##  1     estimate     SE  df t.ratio p.value
##  M - L   -0.275 0.0671 191  -4.105  0.0002
##  M - N   -0.447 0.0794 192  -5.631  <.0001
##  L - N   -0.172 0.0687 189  -2.499  0.0354
##
## Degrees-of-freedom method: satterthwaite
## Results are given on the log10 (not the response) scale.
## P value adjustment: tukey method for comparing a family of 3 estimates
```

Data S2: Full LMM model output comparing whether a plant species is used as a tool source (tool_source) or not may be driven by *EI*, related to Figure 3a and STAR Methods.

```r
# Source model
source_model= lmer(log10(EI) ~ tool_source + log10(I) +(1|plant), data=data)
summary(source_model)
```

```
## Linear mixed model fit by REML. t-tests use Satterthwaite's method [
## lmerModLmerTest]
## Formula: log10(EI) ~ tool_source + log10(I) + (1 | plant)
##    Data: data
##
## REML criterion at convergence: 353.7
##
## Scaled residuals:
##     Min      1Q  Median      3Q     Max
## -3.0081 -0.4290  0.0328  0.4016  4.3104
##
## Random effects:
##  Groups   Name        Variance Std.Dev.
##  plant    (Intercept) 0.13624  0.3691
##  Residual             0.05255  0.2292
## Number of obs: 544, groups:  plant, 194
##
## Fixed effects:
##                Estimate Std. Error        df t value Pr(>|t|)
```

```
## (Intercept)    4.15298    0.43826 538.01882   9.476  < 2e-16 ***
## tool_sourceN   0.26062    0.06767 189.83570   3.852  0.00016 ***
## log10(I)       0.56268    0.03445 538.68921  16.332  < 2e-16 ***
## ---
## Signif. codes:  0 '***' 0.001 '**' 0.01 '*' 0.05 '.' 0.1 ' ' 1
##
## Correlation of Fixed Effects:
##             (Intr) tl_srN
## tool_sourcN -0.216
## log10(I)     0.997 -0.180
```

```
# Source model post-hoc
anova(source_model, type = 2)
```

```
## Type II Analysis of Variance Table with Satterthwaite's method
##               Sum Sq Mean Sq NumDF  DenDF F value    Pr(>F)
## tool_source  0.7795  0.7795     1 189.84  14.834 0.0001604 ***
## log10(I)    14.0165 14.0165     1 538.69 266.739 < 2.2e-16 ***
## ---
## Signif. codes:  0 '***' 0.001 '**' 0.01 '*' 0.05 '.' 0.1 ' ' 1
```

```
emmeans(source_model, list(pairwise ~ tool_source), adjust = 'tukey', lmer.df
= 'satterthwaite')
```

```
## $`emmeans of tool_source`
##  tool_source emmean     SE  df lower.CL upper.CL
##  Y            -2.71 0.0368 244    -2.78    -2.63
##  N            -2.45 0.0582 183    -2.56    -2.33
##
## Degrees-of-freedom method: satterthwaite
## Results are given on the log10 (not the response) scale.
## Confidence level used: 0.95
##
## $`pairwise differences of tool_source`
##  1     estimate     SE  df t.ratio p.value
##  Y - N   -0.261 0.0677 190  -3.852  0.0002
##
## Degrees-of-freedom method: satterthwaite
## Results are given on the log10 (not the response) scale.
```

Data S3: Full LMM model output comparing whether low *EI* was associated with whether an individual plant (plant_used) was actually used to construct a tool or not, related to Figure 3c and STAR Methods.

```
# Used model
used_model= lmer(log10(EI) ~ plant_used + log10(I) +(1|plant), data=data_sele
cted)
summary(used_model)
```

```
## Linear mixed model fit by REML. t-tests use Satterthwaite's method [
## lmerModLmerTest]
## Formula: log10(EI) ~ plant_used + log10(I) + (1 | plant)
##    Data: data_selected
##
## REML criterion at convergence: 291.4
##
## Scaled residuals:
##     Min      1Q  Median      3Q     Max
## -2.5636 -0.3990  0.0423  0.4105  4.1847
##
## Random effects:
##  Groups   Name        Variance Std.Dev.
##  plant    (Intercept) 0.13875  0.3725
##  Residual             0.05648  0.2376
## Number of obs: 408, groups:  plant, 148
##
## Fixed effects:
##              Estimate Std. Error        df t value Pr(>|t|)
## (Intercept)   3.96419    0.53506 404.96020   7.409 7.48e-13 ***
## plant_usedN   0.36807    0.07678 165.75964   4.794 3.62e-06 ***
## log10(I)      0.57009    0.04163 404.65630  13.694  < 2e-16 ***
## ---
## Signif. codes:  0 '***' 0.001 '**' 0.01 '*' 0.05 '.' 0.1 ' ' 1
##
## Correlation of Fixed Effects:
##             (Intr) plnt_N
## plant_usedN -0.155
## log10(I)     0.992 -0.045
```

```
# Used model post-hoc
anova(used_model, type = 2)
```

```
## Type II Analysis of Variance Table with Satterthwaite's method
##             Sum Sq Mean Sq NumDF  DenDF F value    Pr(>F)
## plant_used  1.2979  1.2979     1 165.76  22.982 3.617e-06 ***
## log10(I)   10.5904 10.5904     1 404.66 187.519 < 2.2e-16 ***
## ---
## Signif. codes:  0 '***' 0.001 '**' 0.01 '*' 0.05 '.' 0.1 ' ' 1
```

```
emmeans(used_model, list(pairwise ~ plant_used), adjust = 'tukey', lmer.df =
'satterthwaite')
```

```
## $`emmeans of plant_used`
##  plant_used emmean     SE  df lower.CL upper.CL
##  Y           -3.10 0.0693 174    -3.23    -2.96
##  N           -2.73 0.0393 159    -2.81    -2.65
##
## Degrees-of-freedom method: satterthwaite
## Results are given on the log10 (not the response) scale.
## Confidence level used: 0.95
```

```
##
## $`pairwise differences of plant_used`
##  1     estimate     SE  df t.ratio p.value
##  Y - N   -0.368 0.0768 166  -4.794  <.0001
##
## Degrees-of-freedom method: satterthwaite
## Results are given on the log10 (not the response) scale.
```

Data S4: Full LMM output comparing *EI* of each raw material type (raw_material), related to Figure 3d and STAR Methods.

```
# Raw material model
material_model= lmer(log10(EI) ~ raw_material + log10(I)+(1|plant), data=data
_selected)
summary(material_model)
```

```
## Linear mixed model fit by REML. t-tests use Satterthwaite's method [
## lmerModLmerTest]
## Formula: log10(EI) ~ raw_material + log10(I) + (1 | plant)
##    Data: data_selected
##
## REML criterion at convergence: 142.6
##
## Scaled residuals:
##     Min      1Q  Median      3Q     Max
## -2.4939 -0.5158 -0.0124  0.4412  4.2514
##
## Random effects:
##  Groups   Name        Variance Std.Dev.
##  plant    (Intercept) 0.03382  0.1839
##  Residual             0.05664  0.2380
## Number of obs: 408, groups:  plant, 148
##
## Fixed effects:
##                  Estimate Std. Error        df t value Pr(>|t|)
## (Intercept)       3.70083    0.43384 350.85868   8.530 4.43e-16 ***
## raw_materialV    -0.17619    0.09700 120.66508  -1.816   0.0718 .
## raw_materialB    -0.81651    0.04458 146.04865 -18.316  < 2e-16 ***
## raw_materialG    -0.27947    0.12259 137.58627  -2.280   0.0242 *
## log10(I)          0.50779    0.03436 351.82336  14.778  < 2e-16 ***
## ---
## Signif. codes:  0 '***' 0.001 '**' 0.01 '*' 0.05 '.' 0.1 ' ' 1
##
## Correlation of Fixed Effects:
##             (Intr) rw_mtV rw_mtB rw_mtG
## raw_materlV 0.148
## raw_materlB 0.030  0.143
```

```
## raw_materlG 0.280  0.095  0.123
## log10(I)    0.998  0.161  0.060  0.292

# Raw material model post-hoc
anova(material_model, type = 2)

## Type II Analysis of Variance Table with Satterthwaite's method
##              Sum Sq Mean Sq NumDF  DenDF F value    Pr(>F)    
## raw_material 19.038  6.3462     3 133.45  112.04 < 2.2e-16 ***
## log10(I)     12.371 12.3706     1 351.82  218.40 < 2.2e-16 ***
## ---
## Signif. codes:  0 '***' 0.001 '**' 0.01 '*' 0.05 '.' 0.1 ' ' 1

emmeans(material_model, list(pairwise ~ raw_material), adjust = 'tukey', lmer
.df = 'satterthwaite')

## $`emmeans of raw_material`
##  raw_material emmean     SE  df lower.CL upper.CL
##  T             -2.59 0.0251 144    -2.64    -2.54
##  V             -2.77 0.0955 124    -2.95    -2.58
##  B             -3.41 0.0388 157    -3.48    -3.33
##  G             -2.87 0.1230 143    -3.11    -2.63
## 
## Degrees-of-freedom method: satterthwaite 
## Results are given on the log10 (not the response) scale. 
## Confidence level used: 0.95 
## 
## $`pairwise differences of raw_material`
##  1     estimate     SE  df t.ratio p.value
##  T - V    0.176 0.0970 121   1.816  0.2707
##  T - B    0.817 0.0446 146  18.316  <.0001
##  T - G    0.279 0.1230 138   2.280  0.1078
##  V - B    0.640 0.1010 124   6.352  <.0001
##  V - G    0.103 0.1490 124   0.694  0.8993
##  B - G   -0.537 0.1250 139  -4.289  0.0002
## 
## Degrees-of-freedom method: satterthwaite 
## Results are given on the log10 (not the response) scale. 
## P value adjustment: tukey method for comparing a family of 4 estimates
```

Data S5: Full LMM output comparing whether chimpanzee plant species preference (preference) is driven by *E*, related to Figure 4b and STAR Methods.

```
# Preference model
pref_model_E= lmer(log10(E) ~ preference +(1|plant), data=data)
summary(pref_model_E)

## Linear mixed model fit by REML. t-tests use Satterthwaite's method [
## lmerModLmerTest]
```

```
## Formula: log10(E) ~ preference + (1 | plant)
##    Data: data
##
## REML criterion at convergence: 340.5
##
## Scaled residuals:
##     Min      1Q  Median      3Q     Max
## -3.2807 -0.5034  0.0578  0.5353  3.8840
##
## Random effects:
##  Groups   Name        Variance Std.Dev.
##  plant    (Intercept) 0.19411  0.4406
##  Residual             0.04322  0.2079
## Number of obs: 544, groups:  plant, 194
##
## Fixed effects:
##             Estimate Std. Error        df t value Pr(>|t|)
## (Intercept)  0.52966    0.06606 192.90579   8.018 1.01e-13 ***
## preferenceL  0.24176    0.08059 191.28717   3.000  0.00306 **
## preferenceN  0.28851    0.09443 189.08410   3.055  0.00257 **
## ---
## Signif. codes:  0 '***' 0.001 '**' 0.01 '*' 0.05 '.' 0.1 ' ' 1
##
## Correlation of Fixed Effects:
##             (Intr) prfrnL
## preferenceL -0.820
## preferenceN -0.700  0.573
```

```
# Preference model post-hoc
anova(pref_model_E, type = 2)
```

```
## Type II Analysis of Variance Table with Satterthwaite's method
##             Sum Sq Mean Sq NumDF  DenDF F value   Pr(>F)
## preference 0.50376 0.25188     2 188.86  5.8276 0.003501 **
## ---
## Signif. codes:  0 '***' 0.001 '**' 0.01 '*' 0.05 '.' 0.1 ' ' 1
```

```
emmeans(pref_model_E, list(pairwise ~ preference), adjust = 'tukey', lmer.df
= 'satterthwaite')
```

```
## $`emmeans of preference`
##  preference emmean     SE  df lower.CL upper.CL
##  M           0.530 0.0661 193    0.399    0.660
##  L           0.771 0.0462 188    0.680    0.862
##  N           0.818 0.0675 186    0.685    0.951
##
## Degrees-of-freedom method: satterthwaite
## Results are given on the log10 (not the response) scale.
## Confidence level used: 0.95
##
## $`pairwise differences of preference`
```

```
##  1     estimate     SE  df t.ratio p.value
##  M - L  -0.2418 0.0806 191  -3.000  0.0086
##  M - N  -0.2885 0.0944 189  -3.055  0.0072
##  L - N  -0.0468 0.0818 186  -0.572  0.8353
##
## Degrees-of-freedom method: satterthwaite
## Results are given on the log10 (not the response) scale.
## P value adjustment: tukey method for comparing a family of 3 estimates
```

Data S6: Full LMM output comparing whether a plant species is used as a tool source (tool_source) or not may be driven by *E*, related to Figure 4a and STAR Methods.

```
#Source Model
source_model_E= lmer(log10(E) ~ tool_source +(1|plant), data=data)
summary(source_model_E)

## Linear mixed model fit by REML. t-tests use Satterthwaite's method [
## lmerModLmerTest]
## Formula: log10(E) ~ tool_source + (1 | plant)
##    Data: data
##
## REML criterion at convergence: 346.2
##
## Scaled residuals:
##     Min      1Q  Median      3Q     Max
## -3.3299 -0.4894  0.0542  0.5360  3.8922
##
## Random effects:
##  Groups   Name        Variance Std.Dev.
##  plant    (Intercept) 0.20270  0.4502
##  Residual             0.04325  0.2080
## Number of obs: 544, groups:  plant, 194
##
## Fixed effects:
##               Estimate Std. Error        df t value Pr(>|t|)
## (Intercept)    0.69204    0.03860 190.21306  17.929   <2e-16 ***
## tool_sourceN   0.12603    0.07893 187.17187   1.597    0.112
## ---
## Signif. codes:  0 '***' 0.001 '**' 0.01 '*' 0.05 '.' 0.1 ' ' 1
##
## Correlation of Fixed Effects:
##             (Intr)
## tool_sourcN -0.489

#Source model post-hoc
anova(source_model_E, type = 2)
```

```
## Type II Analysis of Variance Table with Satterthwaite's method
##               Sum Sq Mean Sq NumDF  DenDF F value Pr(>F)
## tool_source 0.11027 0.11027     1 187.17  2.5498  0.112
```

```
emmeans(source_model_E, list(pairwise ~ tool_source), adjust = 'tukey', lmer.
df = 'satterthwaite')
```

```
## $`emmeans of tool_source`
##  tool_source emmean     SE  df lower.CL upper.CL
##  Y            0.692 0.0386 190    0.616    0.768
##  N            0.818 0.0688 186    0.682    0.954
##
## Degrees-of-freedom method: satterthwaite
## Results are given on the log10 (not the response) scale.
## Confidence level used: 0.95
##
## $`pairwise differences of tool_source`
##  1     estimate     SE  df t.ratio p.value
##  Y - N   -0.126 0.0789 187  -1.597  0.1120
##
## Degrees-of-freedom method: satterthwaite
## Results are given on the log10 (not the response) scale.
```

Data S7: Full LMM output comparing whether low *E* was associated with if an individual plant was actually used to construct a tool (plant_used) or not, related to Figure 4c and STAR Methods.

```
# Used model
used_model_E= lmer(log10(E) ~ plant_used +(1|plant), data=data_selected)
summary(used_model_E)
```

```
## Linear mixed model fit by REML. t-tests use Satterthwaite's method [
## lmerModLmerTest]
## Formula: log10(E) ~ plant_used + (1 | plant)
##    Data: data_selected
##
## REML criterion at convergence: 266.7
##
## Scaled residuals:
##     Min      1Q  Median      3Q     Max
## -3.2488 -0.4991  0.0492  0.5247  3.8995
##
## Random effects:
##  Groups   Name        Variance Std.Dev.
##  plant    (Intercept) 0.20515  0.4529
##  Residual             0.04348  0.2085
## Number of obs: 408, groups:  plant, 148
##
## Fixed effects:
```

```
##               Estimate Std. Error        df t value Pr(>|t|)
## (Intercept)   0.46619    0.07764 172.26314   6.005 1.11e-08 ***
## plant_usedN   0.29511    0.08785 178.11317   3.359 0.000956 ***
## ---
## Signif. codes:  0 '***' 0.001 '**' 0.01 '*' 0.05 '.' 0.1 ' ' 1
##
## Correlation of Fixed Effects:
##             (Intr)
## plant_usedN -0.866
```

```
# Used model post-hoc
anova(used_model_E, type = 2)
```

```
## Type II Analysis of Variance Table with Satterthwaite's method
##             Sum Sq Mean Sq NumDF  DenDF F value    Pr(>F)
## plant_used 0.49061 0.49061     1 178.11  11.283 0.0009565 ***
## ---
## Signif. codes:  0 '***' 0.001 '**' 0.01 '*' 0.05 '.' 0.1 ' ' 1
```

```
emmeans(used_model_E, list(pairwise ~ plant_used), adjust = 'tukey', lmer.df
= 'satterthwaite')
```

```
## $`emmeans of plant_used`
##  plant_used emmean     SE  df lower.CL upper.CL
##  Y           0.466 0.0776 172    0.313    0.619
##  N           0.761 0.0440 149    0.674    0.848
##
## Degrees-of-freedom method: satterthwaite
## Results are given on the log10 (not the response) scale.
## Confidence level used: 0.95
##
## $`pairwise differences of plant_used`
##  1     estimate     SE  df t.ratio p.value
##  Y - N   -0.295 0.0879 178  -3.359  0.0010
##
## Degrees-of-freedom method: satterthwaite
## Results are given on the log10 (not the response) scale.
```

Data S8: Full LMM output comparing the *E* of each raw material type (raw_material), related to Figure 4d and STAR Methods.

```
# Raw material model
material_model_E= lmer(log10(E) ~ raw_material +(1|plant), data=data_selected
)
summary(material_model_E)
```

```
## Linear mixed model fit by REML. t-tests use Satterthwaite's method [
## lmerModLmerTest]
## Formula: log10(E) ~ raw_material + (1 | plant)
```

```
##    Data: data_selected
##
## REML criterion at convergence: 182.6
##
## Scaled residuals:
##     Min      1Q  Median      3Q     Max
## -3.0746 -0.5165  0.0282  0.5459  3.8932
##
## Random effects:
##  Groups   Name        Variance Std.Dev.
##  plant    (Intercept) 0.10501  0.3241
##  Residual             0.04367  0.2090
## Number of obs: 408, groups:  plant, 148
##
## Fixed effects:
##                Estimate Std. Error        df t value Pr(>|t|)
## (Intercept)     0.90104    0.03575 134.49248  25.205   <2e-16 ***
## raw_materialV   0.05400    0.14534 129.63811   0.372    0.711
## raw_materialB  -0.75395    0.06502 142.14420 -11.596   <2e-16 ***
## raw_materialG   0.06887    0.17655 131.04282   0.390    0.697
## ---
## Signif. codes:  0 '***' 0.001 '**' 0.01 '*' 0.05 '.' 0.1 ' ' 1
##
## Correlation of Fixed Effects:
##             (Intr) rw_mtV rw_mtB
## raw_materlV -0.246
## raw_materlB -0.550  0.135
## raw_materlG -0.202  0.050  0.111
```

```
# Raw material model post-hoc
anova(material_model_E, type = 2)
```

```
## Type II Analysis of Variance Table with Satterthwaite's method
##              Sum Sq Mean Sq NumDF  DenDF F value    Pr(>F)
## raw_material 6.1543  2.0514     3 134.07  46.977 < 2.2e-16 ***
## ---
## Signif. codes:  0 '***' 0.001 '**' 0.01 '*' 0.05 '.' 0.1 ' ' 1
```

```
emmeans(material_model_E, list(pairwise ~ raw_material), adjust = 'tukey', lm
er.df = 'satterthwaite')
```

```
## $`emmeans of raw_material`
##  raw_material emmean     SE  df lower.CL upper.CL
##  T             0.901 0.0357 134   0.8303    0.972
##  V             0.955 0.1410 129   0.6763    1.234
##  B             0.147 0.0543 146   0.0398    0.254
##  G             0.970 0.1730 131   0.6279    1.312
##
## Degrees-of-freedom method: satterthwaite
## Results are given on the log10 (not the response) scale.
## Confidence level used: 0.95
```

```
##
## $`pairwise differences of raw_material`
##  1     estimate    SE  df t.ratio p.value
##  T - V  -0.0540 0.145 130  -0.372  0.9824
##  T - B   0.7540 0.065 142  11.596  <.0001
##  T - G  -0.0689 0.177 131  -0.390  0.9798
##  V - B   0.8080 0.151 131   5.351  <.0001
##  V - G  -0.0149 0.223 130  -0.067  0.9999
##  B - G  -0.8228 0.181 132  -4.540  0.0001
##
## Degrees-of-freedom method: satterthwaite
## Results are given on the log10 (not the response) scale.
## P value adjustment: tukey method for comparing a family of 4 estimates
```
